# Comparison and Verification of Three Algorithms for Accuracy Improvement of Quartz Resonant Pressure Sensors

**DOI:** 10.3390/mi15010023

**Published:** 2023-12-22

**Authors:** Bin Yao, Yanbo Xu, Junming Jing, Wenjun Zhang, Yuzhen Guo, Zengxing Zhang, Shiqiang Zhang, Jianwei Liu, Chenyang Xue

**Affiliations:** 1 Key Laboratory of Instrumentation Science & Dynamic Measurement, Ministry of Education, North University of China, Taiyuan 030051, China; s202106040@st.nuc.edu.cn (B.Y.); b20230616@st.nuc.edu.cn (Y.G.); sz202206092@st.nuc.edu.cn (S.Z.); s202206098@st.nuc.edu.cn (J.L.); 2 Pen-Tung Sah Institute of Micro-Nano Science and Technology, Xiamen University, Xiamen 361102, China; xuyanbo@stu.xmu.edu.cn (Y.X.); junmin-jing@outlook.com (J.J.); zhangwenjun@stu.xmu.edu.cn (W.Z.); 3 Tan Kah Kee Innovation Laboratory, Xiamen 361005, China

**Keywords:** resonant pressure sensor, multivariate polynomial regression, multilayer perceptron networks, support vector regression, temperature compensation

## Abstract

Pressure measurement is of great importance due to its wide range of applications in many fields. AT-cut quartz, with its exceptional precision and durability, stands out as an excellent pressure transducer due to its superior accuracy and stable performance over time. However, its intrinsic temperature dependence significantly hinders its potential application in varying temperature environments. Herein, three different learning algorithms (i.e., multivariate polynomial regression, multilayer perceptron networks, and support vector regression) are elaborated in detail and applied to establish the prediction models for compensating the temperature effect of the resonant pressure sensor, respectively. The AC-cut quartz, which is sensitive to temperature variations, is paired with the AT-cut quartz, providing the essential temperature information. The output frequencies derived from the AT-cut and AC-cut quartzes are selected as input data for these learning algorithms. Through experimental validation, all three methods are effective, and a remarkable improvement in accuracy can be achieved. Among the three methods, the MPR model has exceptionally high accuracy in predicting pressure. The calculated residual error over the temperature range of −10–40 °C is less than 0.008% of 40 MPa full scale (FS). An intelligent automatic compensation and real-time processing system for the resonant pressure sensor is developed as well, which may contribute to improving the efficiency in online calibration and large-scale industrialization. This paper paves a promising way for the temperature compensation of resonant pressure sensors.

## 1. Introduction

Technological innovations, amplified automation in industries, and a rising need for precise and real-time pressure monitoring solutions have all fueled the substantial growth in the global pressure sensors market. Pressure sensors play a critical role in a variety of industries, including automation, healthcare, aerospace, oil/gas exploitation, and consumer electronics. The aerospace and automotive industries are calling for pressure sensors that can withstand harsh environments and extreme conditions, leading to a surge in demand for these specialized sensors [1]. Quartz resonant sensors have risen as a strong competitor in pressure measurement, showcasing their potential and promise for delivering precise and accurate readings under extreme environments. The remarkable mechanical and electrical features of quartz crystals, such as their high quality factor, compressive strength, and elasticity, are what make them stand out from other pressure sensors, like silicon resonant or silicon piezoresistive [2,3]. These exceptional features of quartz resonant pressure sensors grant them unrivaled authority in scenarios characterized by high temperatures and pressures. 

The AT-cut was discovered in 1934 by Messrs. F.R. Lack and G. W. Willard while working at Bell Labs. It refers to a specific crystalline orientation of anisotropic material quartz with an angle of θ = 35.25° [4]. Experimental verification demonstrated that the AT-cut quartz resonant sensors boast significant pressure sensitivity in pressure measurement applications thanks to the intrinsic electromechanical coupling property of quartz [5,6]. However, they exhibit temperature dependency on frequency, showing a theoretical cubic behavior with a maximum deviation of ±10 ppm in the temperature range from −10 °C to 40 °C due to thermal expansion [7]. This would detrimentally affect the frequency stability and significantly hinder its applicability in the field of high-precision pressure measurement. It is important to improve the pressure sensitivity over a broad range of temperatures to extend the range of applications and minimize the effect of inherent temperature sensitivity on accuracy. Therefore, development of a temperature compensation solution for resonant pressure sensors is crucial for enhancing the overall system performance, enabling them to operate effectively in harsh environments, such as tsunami forecasting and deep-sea diving, for protecting human lives and property. 

Temperature drift compensation methods have been widely studied for decades. They are mainly classified into two categories: passive compensation and active compensation. The former is described from the aspect of device design and encompasses two facets:

- Optimizing the geometry and topology of the sensor structure in order to limit the dependence of thermal expansion behavior on materials’ elastic property [8];
- Adding a composite structure (such as a SiO_2_ layer) to the resonator substrate to obtain an overall satisfactory temperature coefficient [9].

The active compensation method is described from the perspective of signal processing and involves the following points:

- Fabricating temperature-sensitive circuitry to generate temperature-dependent electrical signals, which are used by high-precision logic devices or ASIC to compensate for the output signals from the resonator, thus reducing the temperature drift of the sensor [10,11];
- Establishing the mapping relationship between the physical parameters, i.e., pressure and sensor signals, as well as the temperature information, which is known as multiple regression analysis in statistics [12,13].

Despite the potential for passive compensation methods to effectively mitigate spurious disturbance at a reasonable cost, their fabrication and implementation can be complex and often yield unsatisfactory outcomes. Enhancing the frequency stability of a crystal oscillator can be achieved through temperature compensation circuits, although this approach remains restricted and less efficient compared to the widely accepted regression algorithms that offer greater practicality, precision, and versatility.

The regression algorithms fall into three broad types: machine learning techniques represented by the multilayer perceptron (MLP) network, the statistical-learning-theory-based approach, such as support vector regression (SVR), and the function approximation solution in numerical analysis. Machine learning was ignored for many years due to limitations in computational power. However, it has experienced a revival in recent years due to the availability of a high amount of data. The interactivity and flexibility of artificial neural networks (ANNs) enable it to model intricate relationships with data, adaptively formulating an arbitrary nonlinear function approximation to fit a target value and sensor signals [14]. A new approach to predicting pump output performance using machine learning algorithms has been proposed by Mao [15], with the MLP model showing remarkable accuracy in forecasting pressure and flow. Unlike machine learning, statistical learning devises novel methods to accurately characterize the sensor response and make inferences based on a subset of statistical data gathered during the calibration process [16,17]. As the temperature drift response presents an empirical cubic curve, an intuitive solution is based on function approximation. The function approximation in numerical analysis, termed as least squares (LS) regression, aims to find a linear combination of spline functions (such as polynomials) that transforms the predictors and obtains a multiplicative model to characterize the relationship with data. 

In this work, a comparative study of the three aforementioned regression techniques is carried out based on a pressure sensor using an AT-cut crystal blank. The performance of these algorithms in temperature compensation is determined by the absolute error observed at 40 MPa full scale (FS) across the temperature range of −10–40 °C. The algorithms are executed on a personal computer (PC), and the calculated parameters are then registered into the field-programmable gate array (FPGA) to compensate for the temperature drift. The main purpose of this study is to shed light on the research that uses software methods for sensor temperature compensation and increasing the sensor’s accuracy.

## 2. Materials and Methods

### 2.1. Quartz Resonant Pressure Sensor

A plate-like AT-cut quartz crystal resonator vibrating in thickness-shear mode can be used to measure the applied pressure along the X crystallographic direction [18]. The vibration frequency of the resonator in the thickness-shear mode is provided by

(1) $$f_{n}=\frac{n}{2t}\sqrt{\frac{C_{ij}}{\rho }}\left(n=1,3,5\ldots \right)$$

where 

$f_{n}=$ *n*th resonance frequency;

$C_{ij}=$ elastic modulus for the rotated cut;

t= thickness of the resonator;

ρ= density of the resonator.

It is known that the primary cause of frequency change is due to the change in $C_{ij}$ with respect to the radial stress σ, i.e.,

(2) $$∆f=\frac{f}{2C_{ij}}\times \frac{dC_{ij}}{d\sigma }\times \sigma$$

The resonator’s radial stress, induced by external pressure, causes a proportional variation in its resonant frequency, granting us to detect pressure changes by measuring the frequency shift [19].

**Figure 1:**
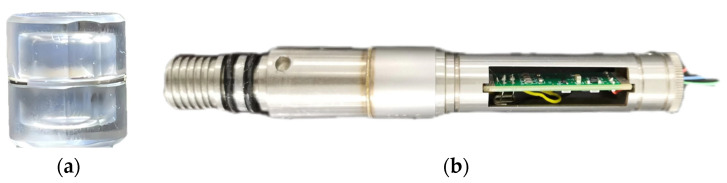
High-precision quartz pressure sensor and packaging. (**a**) Fabricated AT-cut quartz base pressure sensor device. Two quartz-based end caps bond the resonator through glass-frit layer. (**b**) Sensors are placed into a stainless housing, where the PCB converts the pressure and temperature signals to frequencies.

The high-precision quartz resonant pressure sensor shown in Figure 1a is fabricated based on an integral part of a quartz cylinder. A single piece of crystalline quartz is used to construct the resonator and cylinder, and, consequently, the periphery of the former will be uniformly and elastically stressed by the external pressure [20]. The cylindrical walls act as a diaphragm, effectively transferring and concentrating the stress from the external pressure radially about the periphery of the resonator [12]. This process attenuates hysteresis errors, unlike the conventional method that applies force with anvils at the resonator’s edge, leading to localized stress concentration that is the primary cause of hysteresis [21]. The quartz end caps are aligned such that the crystalline lattice is essentially continuous across the glass-frit sealing layer, ensuring the system’s valid continuum mechanics under external forces [22]. The cavity herein, which may be empty or helium-filled, isolates the resonator from the pressure media to achieve a high degree of frequency stability and low mechanical damping. The sputtered golden metallic film on both sides of AT-cut crystal surface acts as the resonator electrodes and external electrical connections. The AC-cut crystal, which is temperature-sensitive and placed near the pressure sensor, is employed to detect the ambient temperature. Unlike the existing Pt thin-film temperature sensor, piezoelectric crystal provides advantages for temperature measurement, such as high accuracy, low power consumption, direct processing of the ambient temperature, and, more importantly, increased operating range with respect to temperature [3].

The oscillator circuit maintains resonant vibration of the sensor and outputs a signal whose frequency is directly proportional to the pressure. The output signal of reference oscillator is mixed with the pressure and temperature signals to reduce their frequencies from the MHz range to the kHz range, simplifying the frequency counting requirements. The electronic counter converts the number of counts generated by the sensor in a given gate time to a digital frequency. Figure 1b shows an assembled quartz resonant pressure sensor with stainless housing. The PCB detects the pressure and temperature signals and outputs them in the form of digital frequencies. Note that the packaged sensor size can be further reduced significantly by using a smaller container and integrated circuit instead of the PCB, which can meet requirements of various applications.

**Figure 2:**
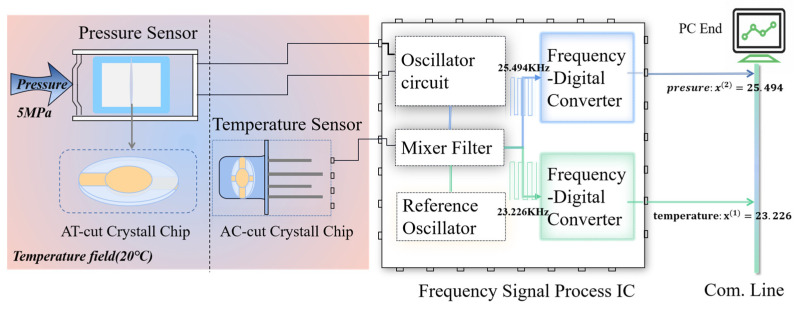
Schematic diagram of pressure detection and calibration system based on quartz devices. Take the ambient temperature of 20 °C and pressure of 5 MPa for instance.

Figure 2 shows the schematic diagram of pressure and temperature signals processing. The digital signals are acquired by a laptop for signal analysis, calibration, and processing for temperature compensation. In the figure, $x^{\left(1\right)}$ and $x^{\left(2\right)}$ represent the numerical temperature and pressure information transformed by the frequency-digital converter in the units of kHz, respectively. The methods of utilizing the two pieces of information to calibrate the measured pressure value based on regression algorithms are elaborated thereafter.

### 2.2. Types of Regression Methods

#### 2.2.1. Multivariate Polynomial Regression (MPR)

It is known that, in multivariate tensor product polynomial spaces, stable and accurate discrete LS approximation of any continuous function can be obtained using low-discrepancy point sets [23,24]. Based on this information, we consider a parsimonious linear parametric model to fit the longitudinal data [25], which consist of the sensor’s temperature and pressure frequency observations at varying ambient temperatures, and the actual pressure value y. The purpose of this fitting is to efficiently build a finite dimensional approximation of temperature compensation function, namely f. To this end, we choose one-dimensional monomial bases with respect to each variable $x^{\left(i\right)}$ that indicate the element of sensor information. These bases are shown below [26]:

(3) $${\left\{\phi_{j}^{i}\right\}}_{j=1}^{\infty },i=1,2,\ldots ,d$$

where $\phi_{j}^{i}:={x^{\left(i\right)}}^{j}$ is called the jth order basis function or feature for ith variable that coincides with the temperature frequency (i = 1) or pressure frequency (i = 2) data, with the dimension parameter d equal to 2. Subsequently, the multidimensional bases can be formed by tensorizing the univariate bases ${\left\{\phi_{j}^{i}\right\}}_{j=1}^{\infty }$. To explicitly form these bases, given a basis order q, the multi-index is defined as follows:

(4) $$\Lambda_{P}^{2,q}≔\left\{n=\left(n_{1},n_{2}\right)\in N^{2}:\;\underset{i=1,2}{max}\;\;n_{i}\leq q\right\}$$

As an example, $\Lambda_{P}^{2,2}$ set comprises $\left(0,0\right),\left(0,1\right),\left(0,2\right);\left(1,0\right),\left(1,1\right),\left(1,2\right);\left(2,0\right),\left(2,1\right),\left(2,2\right)$.

Based on a concise and established terminology presented in the literature [27], we denote the finite dimensional multivariate polynomial space as

(5) $$P_{\Lambda }=\mathrm{span}\left\{\Phi_{p}≔\prod_{i=1}^{2}\;\phi_{p_{i}}^{i},\;\mathrm{with}\;p\in \Lambda_{P}^{2,q}\right\}$$

The above expression means that the polynomial feature space is associated with the index set Λ and coefficients in R. The best approximation of f denoted by $P_{\Lambda }f$ in $P_{\Lambda }$ can be written as follows:

(6) $$P_{\Lambda }f=\sum_{p\in \Lambda_{P}^{2,q}}c_{p}\Phi_{p}=\sum_{i=0}^{b}\sum_{j=0}^{d}c_{i,j}\phi_{i}^{1}\phi_{j}^{2}\;\;\;\;\;b,d\leq q$$

(7) $$x\overset{P_{\Lambda }f}{\to }\sum_{i=0}^{b}\sum_{j=0}^{d}c_{i,j}\phi_{i}^{1}{\left(x\right)\phi }_{j}^{2}\left(x\right)=\sum_{i=0}^{b}\sum_{j=0}^{d}c_{i,j}{x^{\left(1\right)}}^{i}{x^{\left(2\right)}}^{j},\;\;\;x=\left(x^{\left(1\right)},x^{\left(2\right)}\right)$$

where $c={\left(c_{0,0},c_{0,1},\ldots ,{c_{0,d},\ldots ,c}_{b,d}\right)}^{T}$ is the coefficient vector. In the framework of discrete LS, the optimal coefficient $c^{*}$ can be determined by solving the following problem:

(8) $$c^{*}=\underset{P_{\Lambda }f\in P_{\Lambda }}{argmin}\sum_{k=1}{\left\|P_{\Lambda }f\left(x_{k}\right)-y_{k}\right\|}^{2}$$

where $\left\|\cdot \right\|$ denotes the Euclidean norm. 

The coefficient $c^{*}$ should be determined over the complete training set $(X,Y)=\left\{\left(x_{k},y_{k}\right),k=1,\ldots ,m\right\}$ of observed data, where $x_{k}$ is the vector form of $\left(x_{k}^{\left(1\right)},x_{kj}^{\left(2\right)}\right),\;j=1,2,\ldots ,n_{k}$; $y_{k}={\left[y_{k1},y_{k2},\ldots ,y_{kn_{k}}\right]}^{T}$, and $n_{k}$ is the number of observations at the kth temperature point. The algebraic formulation using the SVD factorization is

(9) $$\;\left(c_{i,j}\right)=\left(\begin{array}{ccc} c_{0,0} & \cdots & c_{0,d}\\ \vdots & \ddots & \vdots \\ c_{b,0} & \cdots & c_{b,d} \end{array}\right)={\varphi^{\left(1\right)}}^{+}⨂{\left[\left({\varphi_{1}^{\left(2\right)}}^{+},{\varphi_{2}^{\left(2\right)}}^{+},\ldots ,{\varphi_{m}^{\left(2\right)}}^{+}\right)⨂y\right]}^{T}\;={\varphi^{\left(1\right)}}^{+}{\left({\varphi_{1}^{\left(2\right)}}^{+}y_{1},{\varphi_{2}^{\left(2\right)}}^{+}y_{2},\ldots ,{\varphi_{m}^{\left(2\right)}}^{+}y_{m}\right)}^{T}$$

where

(10) $$\;\varphi^{\left(1\right)}=\left(\begin{array}{ccc} 1 & \cdots & {{(x}_{1}^{\left(1\right)})}^{b}\\ \vdots & \ddots & \vdots \\ 1 & \cdots & {{(x}_{m}^{\left(1\right)})}^{b} \end{array}\right);\;\varphi_{k}^{\left(2\right)}=\left(\begin{array}{ccc} 1 & \cdots & {{(x}_{k1}^{\left(2\right)})}^{d}\\ \vdots & \ddots & \vdots \\ 1 & \cdots & {{(x}_{kn_{k}}^{\left(2\right)})}^{d} \end{array}\right),\;k=1,2,\ldots ,m.$$

**Figure 3:**
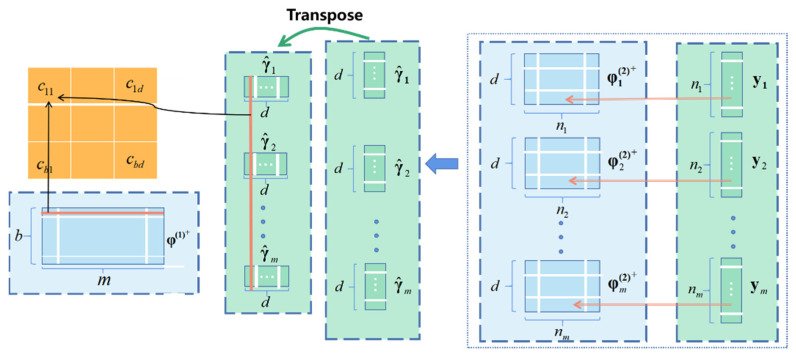
Signal flow diagram in accordance with Equation (9).

The design matrix is denoted by φ, with each row representing an individual observation of a variable. Figure 3 shows the flow diagram of the MPR algorithm. The varying actual pressure values at the same temperature point are formed into a vector denoted by $y_{k}$. Multiplying $y_{k}\;(k=1,2,\ldots ,m)$ by the pressure design matrix $\varphi_{k}^{\left(2\right)}$ that pertains to the pressure frequencies observed under the kth temperature point, m sets of vectors $\left[\gamma_{k}\;\right|k=1,2,\ldots ,m]$ are obtained. The *γ* vector sets are stacked column-wise into a matrix, followed by a transpose operation. Subsequently, we can obtain the coefficient matrix by multiplying the temperature design matrix and the aforementioned transposed matrix.

#### 2.2.2. MLP

Artificial neural network (ANN) is a structure that simulates the essential features of neurons and their interconnections with the aim of processing information [28]. One of the very first ANN architectures is the multilayer perceptron (MLP). Many researchers have employed this parallel distributed type network as a benchmark model [29,30,31]. 

**Figure 4:**
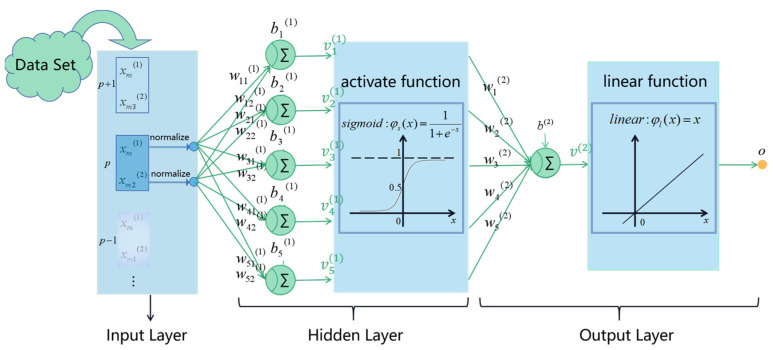
An MLP network, consisting of multiple layers of connected neurons.

The architectural graph shown in Figure 4 illustrates the layout of a multilayer feedforward neural network with a single hidden layer, which has two source nodes whose inputs are temperature and pressure frequency information denoted by $x^{\left(1\right)}$, $x^{\left(2\right)}$, respectively, five hidden neurons, and a single output neuron that provides the predicted pressure value. This network can be described in a compact mathematical formulation as follows:

(11) $$F\left(x;w\right)=\sum_{j=1}^{5}w_{j}^{\left(2\right)}\varphi \left(\sum_{i=0}^{2}w_{ji}^{\left(1\right)}x_{i}\right)+w_{0}^{\left(2\right)}\;$$

Correspondingly, we formulate the model’s cost function as

(12) $$\varepsilon \left(N;w\right)=\frac{1}{2N}\sum_{i=1}^{N}{\left[y\left(n\right)-F\left(x\left(n\right);w\right)\right]}^{2}$$

where $w_{j0}^{\left(1\right)}$ represents the bias of jth neuron in the hidden layer, and $w_{0}^{\left(2\right)}$ is the bias of neuron in the output layer. The nonlinear activation function denoted by φ(·) is required to be continuously differentiable, and commonly used activation functions in MLP are ReLU and Sigmoid functions.

The objective is to select a particular hypothesis $F\left(x;w\right)$ that minimizes the cost function. It is defined as follows:

(13) $$w^{*}=\underset{w}{argmin}\varepsilon \left(N;w\right)$$

**Figure 5:**
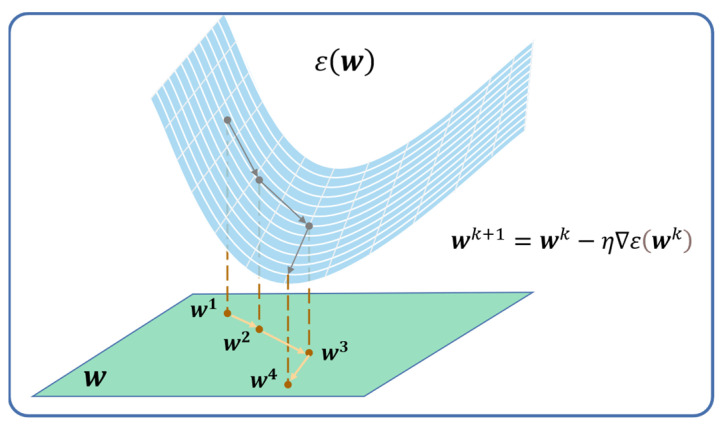
Illustration of the search of the optimum value of w; ε(w) is the cost function defined as the sum of squared errors over N.

The backpropagation (BP) algorithm, which is an efficient application of chain-rule-based supervised learning, is used to optimize the network parameters. It makes the weight vector $w^{k}$ follow a well-defined and deterministic trajectory in weight space computed by the steepest descent method, which uses an instantaneous estimate δ provided by (14) and (15) of the gradient of the error surface to update the network [32], as illustrated in Figure 5. More specifically, the network weights are initialized randomly and then trained consistently through iteratively minimizing the difference between the actual and predicted pressure values. In each training iteration, each weight in the neural network is updated based on the current weight and is proportional to the partial derivative of the error function. The required gradients can be obtained with ease via backpropagating error derivatives through the network using the chain rule. This is the core part of the BP algorithm and can be described by the following equations [33]:

(14) $$for\;output\;layer:\;\;\;\;\;\left\{\begin{array}{c} ∆w_{j}^{\left(2\right)}=-\eta \frac{\partial \varepsilon }{{\partial w}_{j}^{\left(2\right)}}=-\eta \;\frac{\partial \varepsilon }{\partial v^{\left(2\right)}}\;\frac{\partial v^{\left(2\right)}}{{\partial w}_{j}^{\left(2\right)}}=-\eta \delta^{\left(2\right)}\varphi \left(v_{j}^{\left(1\right)}\right)\\ \delta^{\left(2\right)}=-\frac{\partial \varepsilon }{\partial v^{\left(2\right)}}=e \end{array}\right.$$

(15) $$\;\;\;\;\;\;\;for\;hidden\;layer:\;\;\;\;\;\left\{\begin{array}{c} ∆w_{ji}^{\left(1\right)}=-\eta \frac{\partial \varepsilon }{{\partial w}_{ji}^{\left(1\right)}}=-\eta \frac{\partial \varepsilon }{\partial v_{j}^{\left(1\right)}}\;\frac{\partial v_{j}^{\left(1\right)}}{{\partial w}_{ji}^{\left(1\right)}}=-\eta \delta_{j}^{\left(1\right)}x_{i}\\ \delta_{j}^{\left(1\right)}=-\frac{\partial \varepsilon }{\partial v_{j}^{\left(1\right)}}=-\frac{\partial \varepsilon }{\partial v^{\left(2\right)}}\cdot \frac{\partial v^{\left(2\right)}}{\partial v_{j}^{\left(1\right)}}=ew_{j}^{\left(2\right)}\dot{\varphi }\left(v_{j}^{\left(1\right)}\right),j=1,2,\ldots \end{array}\right.$$

where e represents the instantaneous error of the model. The induced local $v_{j}^{\left(1\right)}$ produced at the input of the activation function associated with neuron j from the hidden layer is written as $v_{j}^{\left(1\right)}=\sum_{i=0}^{2}w_{ji}^{\left(1\right)}x_{i}$, and, similarly, $v^{\left(2\right)}=\sum_{j}w_{j}^{\left(2\right)}\varphi (v_{j}^{\left(1\right)})$. The constant η is the learning-rate parameter of the BP algorithm, which decides the step length towards the minimum for each iteration. The weights of MLP are adjusted after all the N samples have been fed forward, which constitutes a training epoch. The BP algorithm is considered to have converged when the mean square error (MSE) per epoch is sufficiently small, or it is stopped when the training epochs reach the target number of epochs.

#### 2.2.3. SVR

Support vector regression (SVR) is a robust and efficient algorithm developed by Vapnik. It is a nonlinear prediction method based on statistical learning theory [34,35]. The compliance of structural risk minimization inductive principle enables the prediction model to obtain good generalization based on a limited number of learning patterns, i.e., the training sample set $\;\left\{(x,y)|\;x∶=\left(x^{\left(1\right)},x^{\left(2\right)}\right)\right\}$, where $x^{\left(1\right)}$,$\;x^{\left(2\right)}$, and y represent the temperature and pressure information from the sensor’s output and the corresponding actual pressure value, respectively.

**Figure 6:**
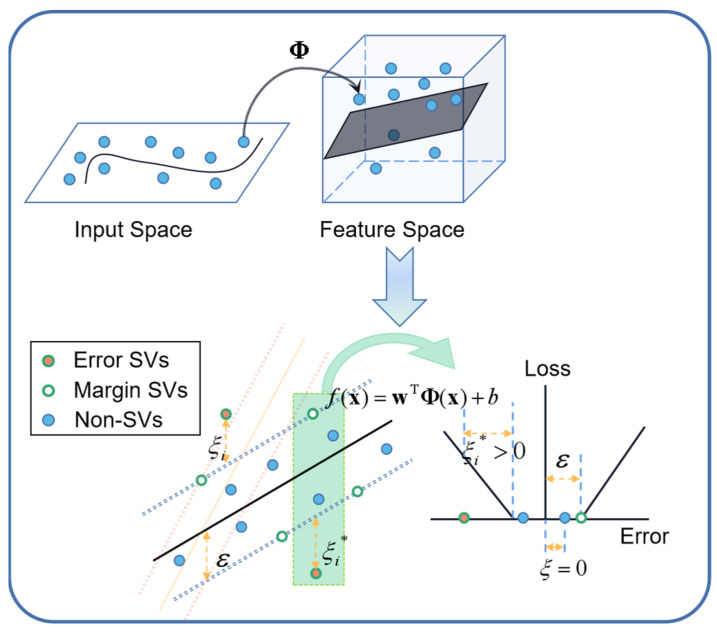
Graphical summary of nonlinear SVR.

According to Cover’s Theorem, a set of training data is linearly separable with a high probability if the data are projected in a feature space of infinite dimensionality based on a set of nonlinear functions ${\left\{\phi_{j}(x)\right\}}_{j=1}^{\infty }$, as shown in the top part of Figure 6. Given this transformation, we may define a hyperplane acting as the decision surface for the regression task in accordance with the following expression [36]:

(16) $$f\left(x\right)=w^{T}\Phi \left(x\right)+b$$

where the feature vector $\Phi \left(x\right)={\left[\phi_{1}\left(x\right),\phi_{2}\left(x\right),\ldots \right]}^{T}$ is infinite, and w and b correspond to the weight vector and bias constant, respectively. 

As shown in (17), SVR works by solving a constrained quadratic problem where the convex function for minimization is provided by the combination of a loss function with a regularization term, which is the norm of the weights [37].

(17) $$\left\{\begin{array}{c} {Minimize:\;\;\;\;L}_{SVR}=\frac{1}{2}{\left\|w\right\|}^{2}+C\sum_{i=1}^{N}\left(\xi_{i}+\xi_{i}^{*}\right)\\ Subject\;to:\;\;\left\{\begin{array}{c} y_{i}-f\left(x_{i}\right)\leq \varepsilon +\xi_{i}\\ f\left(x_{i}\right)-y_{i}\leq \varepsilon +\xi_{i}^{*}\\ \xi_{i},\xi_{i}^{*}\geq 0 \end{array}\right.\;,\;0\leq i\leq N \end{array}\right.$$

The solution to the problem in (17) attempts to minimize the generalized error bound to achieve a generalized performance, which is required to minimize the Euclidean norm, i.e., ${\left\|w\right\|}^{2}$. The ε-insensitive loss illustrated in the bottom part of Figure 6 determines that the prediction function $f\left(x\right)$ has at most a deviation of ε from the target, and all further deviations should incur only a linear penalty. In order to cope with the infeasible constraints of the optimization problem, slack variables $\xi_{i}$ and $\xi_{i}^{*}$ are introduced to evaluate the deviation in training data outside the ε-*insensitive* region. The penalty parameter C>0 provides a tradeoff between the distance of the ε-tube margin and the amount up to which deviations larger than ε are tolerated. We can solve the constrained optimization problem in (17) by using the method of Lagrange multipliers, and the dual form of the problem can be expressed as follows [38]:

(18) $$\left\{\begin{array}{c} Maximize:-\frac{1}{2}\sum_{i=1}^{N}\sum_{j=1}^{N}\left(\alpha_{i}-\alpha_{i}^{*}\right)\left(\alpha_{j}-\alpha_{j}^{*}\right)\langle \Phi \left(x_{i}\right),\Phi \left(x_{j}\right)\rangle -\sum_{i=1}^{N}\varepsilon \left(\alpha_{i}+\alpha_{i}^{*}\right)-\sum_{i=1}^{N}y_{i}\left(\alpha_{i}-\alpha_{i}^{*}\right)\\ Subject\;to:\;\;\sum_{i=1}^{N}\left(\alpha_{i}-\alpha_{i}^{*}\right)=0\;\;,\left(0\leq \alpha_{i},\alpha_{i}^{*}\leq C\right) \end{array}\right.$$

where the auxiliary nonnegative variables $\alpha_{i}$ and $\alpha_{i}^{*}$ represent the Lagrange multipliers, and $\langle \;,\rangle$ denotes the inner product operation. 

As the dimensionality of feature vector $\Phi \left(x\right)$ is infinite, it is impossible to calculate the inner product in the feature space directly. This problem is mitigated by introducing kernel as follows, where it is denoted as the scaler:

(19) $$k\left(x_{i},x_{j}\right)∶=\langle \Phi \left(x_{i}\right),\Phi \left(x_{j}\right)\rangle =\sum_{l=1}^{\infty }\phi_{l}\left(x_{i}\right)\phi_{l}\left(x_{j}\right)$$

Radial basis function (RBF) with the mathematical expression as $k\left(x_{i},x\right)=e^{-\gamma {\left\|x_{i}-x\right\|}^{2}}$ is being increasingly used in machine learning and provides a good choice for the kernel [39]. The expansion of the RBF kernel permits us to construct a decision surface that is nonlinear in the input space but is linear in the feature space. Substituting (19) into (18), and utilizing sequential minimal optimization (SMO) method to compute the dual problem, we obtain the following desired expression for the hyperplane:

(20) $$\left\{\begin{array}{c} f\left(x\right)=\sum_{i=1}^{N_{s}}{\alpha_{i}}^{\left(*\right)}k\left(x_{i},x\right)+b\\ b=y_{l}+\varepsilon -\sum_{i=1}^{N_{s}}{\alpha_{i}}^{\left(*\right)}k\left(x_{i},x_{l}\right) \end{array}\right.$$

where the nonvanishing Lagrange multipliers ${\alpha_{i}}^{\left(*\right)}$ correspond to the support vectors, and $N_{s}$ refers to the number of support vectors. 

## 3. Results

### 3.1. Calibration System and Performance Characteristics before Compensation

**Figure 7:**
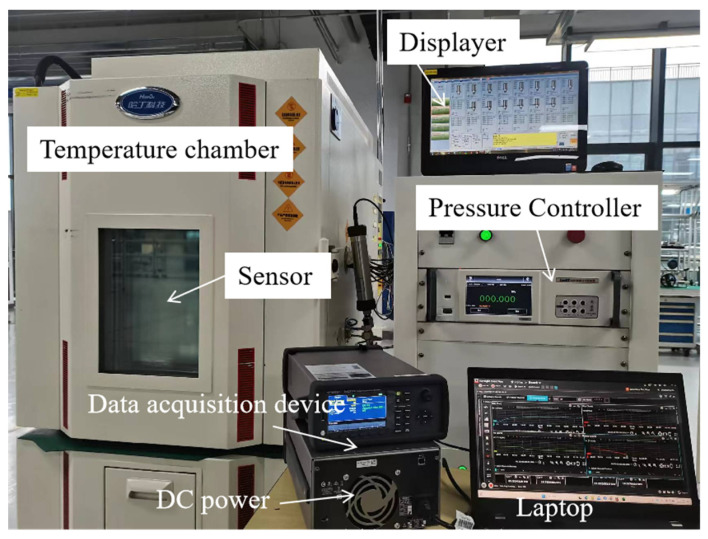
Experimental setup of the static calibration test.

The calibration experiment is carried out at a company named Beijing Hunge Testing Technology Co. Ltd. (Beijing, China), which obtains measurement traceability from the National Metrology Institutes of China. The company furnishes a high-precision piston-type pressure gauge with an accuracy of 0.001% FS over the calibrated temperature range from −10 to 40 °C. In addition, a temperature test chamber with an ambient fluctuation of less than 0.3 °C is utilized. Figure 7 shows the temperature compensation process adopted in the experiment. The pressure controller can automatically regulate the piston pressure gauge and show the current pressure data on the LCD displayer. A data acquisition device is required to measure the output frequency of the sensor. Additionally, the sensor is supplied with 4.5 V DC power. After collecting the calibration data, the regression model for temperature compensation is generated automatically on a laptop. We have developed a software called the CTD Panel 1.0 to facilitate the establishment of the regression model. It is based on the Qt5 framework and coded in Python 3.10 [40] for temperature compensation of the resonant pressure sensor. This software implements the three algorithms investigated in this paper.

The calibration experiment includes six different temperature preservation processes: −10 °C, 0 °C, 10 °C, 20 °C, 30 °C, and 40 °C. The pressure calibration points are selected in an arithmetic sequence over a range of 0 to 40 MPa, tested every 5 MPa over a total of nine pressure points. Thus, a total of 54 datasets are ultimately collected. It is important to minimize the impact of environmental temperature imbalance on pressure frequency output. This is carried out by stabilizing the temperature for approximately 20 min when transitioning between temperature points during the test. Once the fluctuation of the pressure frequency drops below 0.01 Hz per minute, the sensor data are recorded and transmitted to the laptop for subsequent data analysis. 

**Figure 8:**
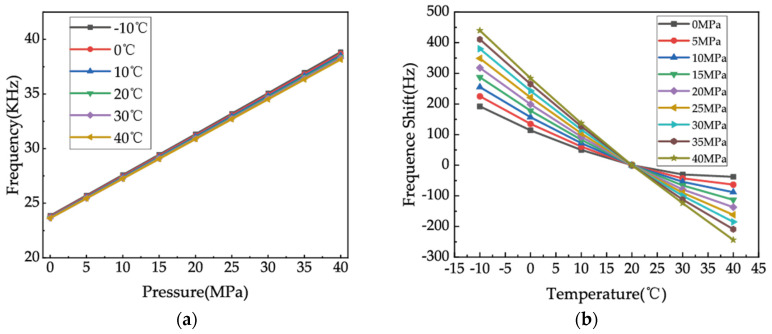
Temperature influence research on the resonant pressure sensor. (**a**) Frequency response under a full pressure range of 0–40 MPa and a temperature range of −10–+40 °C. (**b**) Frequency shift caused by the temperature.

As Figure 8a shows, the preliminary measurements suggest that the fabricated resonant pressure sensor exhibits a strongly linear response when subjected to a constant temperature, demonstrating a sensitivity of 370 Hz/MPa at an ambient temperature of 40 °C. Considering 20 °C as the benchmark, Figure 8b exhibits the frequency drift caused by temperature changes at different constant pressure values. The graphs indicate that the frequency drift phenomenon is more pronounced at higher applied pressures. The output frequency exhibits a variation of 678 Hz upon exposure to a temperature between −10 and 40 °C, while ensuring a consistent applied pressure of 40 MPa. Given the estimated sensitivity of 370 Hz/MPa, the deviation of 678 Hz results in an approximate calibrated pressure error of 1.8 MPa, which significantly exceeds the normal margin of expected error. The introduction of nonlinearity into the pressure signal has caused serious issues for the sensor’s application; thus, it is necessary to compensate for the detrimental effect of nonlinearity caused by temperature by means of appropriate regression algorithms.

### 3.2. Temperature Compensation Using MPR

The order of the multivariate polynomial model is crucial to the prediction accuracy because it directly determines the dimensions of the projected polynomial feature space [41]. As the order increases, the accuracy of the regression model improves because the MPR converges to the unknown function that provides the relationship between the actual pressure, frequencies of the AT-cut vibration, and the temperature information produced by the AC-cut. However, an excessively high order would cause overfitting that is detrimental to the generalization ability of the model. 

**Figure 9:**
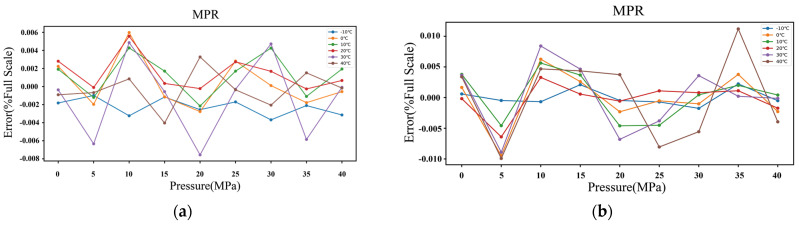
Relative full-scale error versus pressure under (**a**) fifth order, and (**b**) fourth order, respectively.

**Figure 10:**
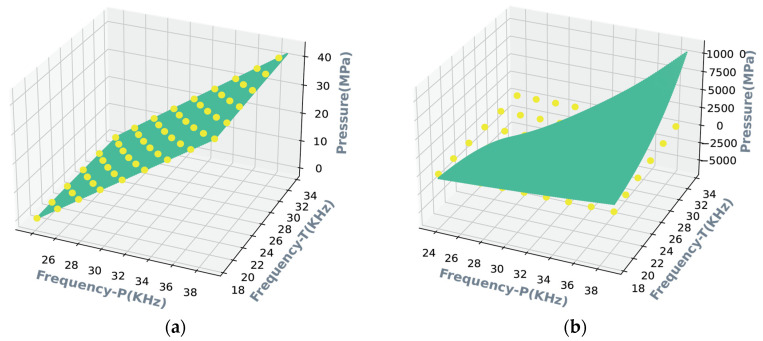
Illustration of the surface as a decision region under (**a**) fifth order, and (**b**) sixth order, respectively. Calibration data is indicated by the yellow dots, and the green areas depict the three-dimensional graphs of the regression function.

**Figure 11:**
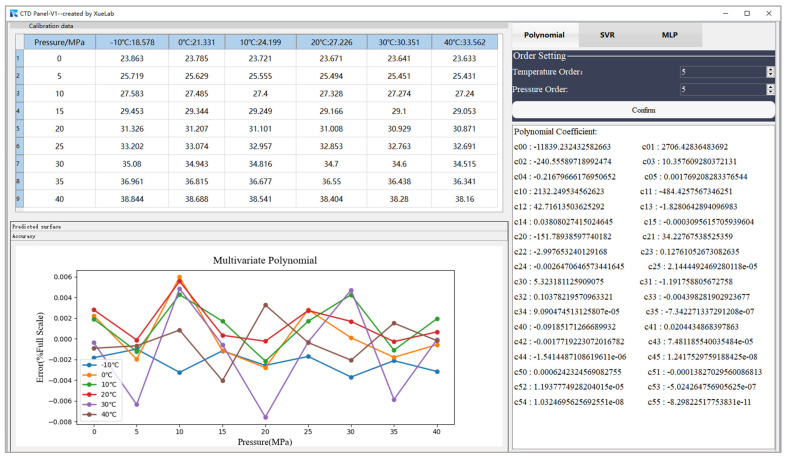
Interface diagram of the CTD Panel as a tool to build a temperature compensation model of MPR for the sensor.

This paper demonstrates experimentally that the optimal accuracy of the multivariate polynomial model can reach ±0.008% FS when the model order is five, as shown in Figure 9a. On the other hand, the accuracy with a fourth model polynomial order degrades sharply to ±0.02% FS, as shown in Figure 9b. Figure 10 shows a map of the decision regions in the two-dimensional signal space spanned by the input variables of pressure and temperature frequency. The decision function takes the form of a smooth green surface, and the calibration data are indicated by yellow circles. It can be noted from Figure 10a that the calibration samples and predictive results match closely. Furthermore, the decision surface is no longer confined to the provided learning samples, as shown in Figure 10b when a higher basis order of six is used. Figure 11 illustrates the use of CTD Panel as an MPR model design tool. Once we have set up any combination of orders assigned to design matrices for temperature and pressure, respectively, the polynomial coefficients are ready to be obtained. The desired algorithm can be formulated based on the following function:

(21) $$f^{*}\left(x\right)=\sum_{i=0}^{5}\sum_{j=0}^{5}c_{i,j}{x^{\left(1\right)}}^{i}{x^{\left(2\right)}}^{j}$$

### 3.3. Temperature Compensation Using MLP

The Universal Approximation Theorem provides the framework for the design of nonlinear systems that can effectively approximate any multivariate continuous function. However, it does not actually specify how to determine an MLP configuration according to a specified approximation accuracy. There is no established theory regarding the optimal number of hidden layers, as well as the number of neurons assigned to each layer. Generally, the MLP model can approximate the unknown function with any desired accuracy provided that a sufficiently large number of hidden units are used at the expense of lower generalization capacity of the networks.

**Figure 12:**
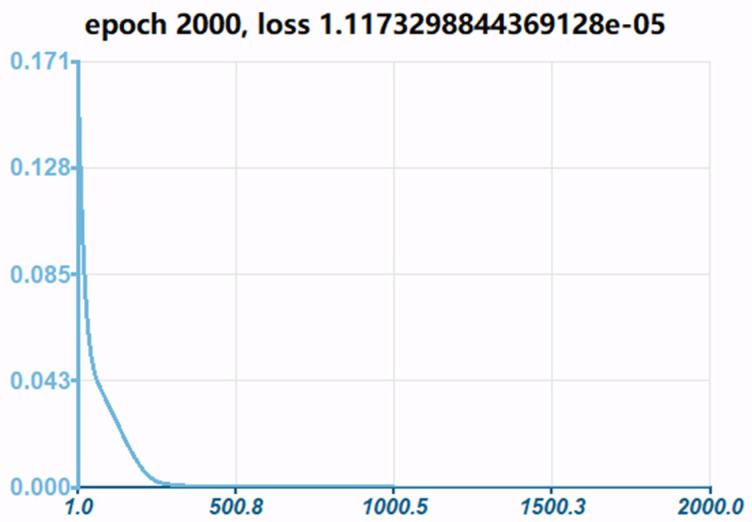
Learning curve within 2000 epochs.

**Figure 13:**
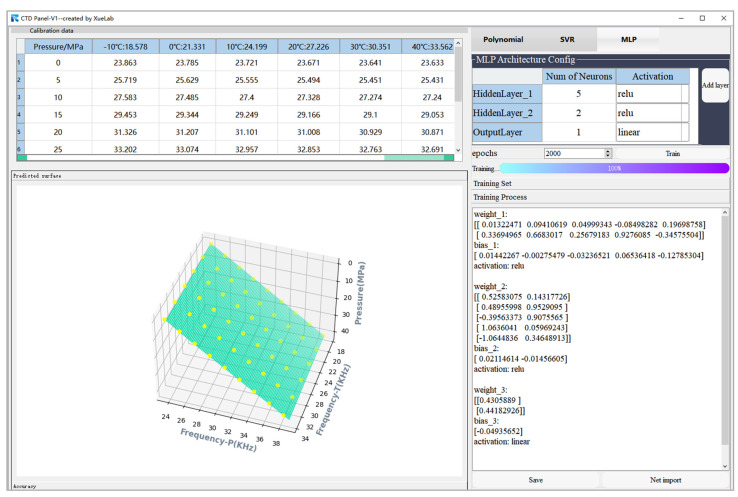
Interface diagram of the CTD Panel used as a tool to build a temperature compensation model of MLP for the sensor. Calibration data is indicated by the yellow dots, and the green areas depict the three-dimensional graphs of the regression function.

Empirically, we implement the MLP networks with two hidden layers as a tradeoff between global optimization (i.e., attempting to avoid being trapped in local minima) and model complexity. Consequently, the number of neurons in the first and second hidden layers are set to five and two, respectively, based on trial and error. ReLU with the mathematical representation as $\varphi \left(x\right)=max(0,x)$ is chosen as the nonlinear activation function in hidden layers, which is attributed to its efficacy in addressing the issue of vanishing gradient. Consider an epoch of the 54 training examples arranged in the order $\left\{x\left(1\right),y\left(1\right)\right\},\;\left\{x\left(2\right),y\left(2\right)\right\},\ldots ,\left\{x\left(54\right),y\left(54\right)\right\}$. In each training epoch, the examples in the training set are randomly shuffled, thereby guaranteeing convergence of the method of steepest descent to a local minimum. Adjustment to the synaptic weights of the MLP occurs on an epoch-by-epoch basis. The total number of training epochs is equal to 2000. Figure 12 shows one realization of the learning curve that is obtained by plotting the loss (MSE) versus the number of epochs, assuming a fixed learning-rate parameter of 0.001. Due to the complicated nature of the error surface, the training process usually tends to zigzag towards the true direction to the minimum on the error surface. Randomly assigning the initial weights of the neurons in the BP algorithm makes it stochastic, leading to different training outcomes even with the same network configuration. Using the CTD Panel neural network design tool, the MLP algorithm can be formulated as shown in Figure 13. The equivalent function of the model can be deduced as

(22) $$\left\{\begin{array}{c} f^{*}\left(x\right)=\sum_{l=1}^{2}w_{l}^{\left(3\right)}\varphi \left(\sum_{j=1}^{11}w_{kj}^{\left(2\right)}\varphi \left(\sum_{i=1}^{2}w_{ji}^{\left(1\right)}x^{\left(i\right)}+b_{j}^{\left(1\right)}\right)+b_{k}^{\left(2\right)}\right)+b_{l}^{\left(3\right)}\\ \varphi \left(x\right)=\max\left(0,x\right) \end{array}\right.$$

**Figure 14:**
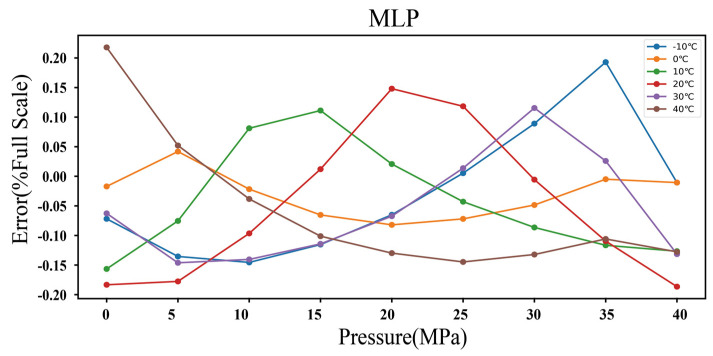
Accuracy of the resonant pressure sensor based on the MLP temperature compensation model.

Figure 14 shows that the accuracy of the resonant pressure sensor conforms to within ±0.3% FS when the MLP is used with 2000 training epochs to compensate for the temperature drift. The low accuracy is caused by the lack of training data, due to which the learned distribution cannot perfectly mirror the real distribution while simultaneously ensuring the model’s ability to generalize. If the number of examples is sufficiently large, we may expect the network to generalize better than what has been demonstrated in this work.

### 3.4. Temperature Compensation Using SVR

The hyperparameters, such as regularization parameter C, insensitivity ε, and kernel parameter γ, should be carefully designed such that the SVR solution achieves a satisfactory balance between accuracy and generalization [42]. Insight about the generalization of SVR can be obtained from the inner-product kernel between a “support vector” $x_{i}$ and a vector x drawn from the input data space. For the previously discussed kernel method such as the RBF, the kernel parameter is usually fixed. The receptive field of the RBF kernel unit, which is used to confine the local average to observations in a small neighborhood, can be defined as follows:

(23) $$\psi \left(x\right)=e^{-\gamma {\left\|x_{i}-x\right\|}^{2}}-a>0$$

where the domain of the input x is confined to a circular disc of $x_{i}$ (i.e., support vector) and radius $\sqrt{\ln\frac{1}{a}/\gamma }$, with a denoting the threshold above which the model generates a response. Therefore, the γ parameter in the RBF kernel function can be viewed as the reciprocal of the influence radius of the support vector. If γ is too small, the receptive field of any selected support vector would tend to include the whole training set, and it is possible for the SVR to underfit the training data. On the other hand, when γ is too large, the receptive field of the support vector would only cover the support vector itself and no amount of regularization with C will be able to prevent overfitting. 

**Figure 15:**
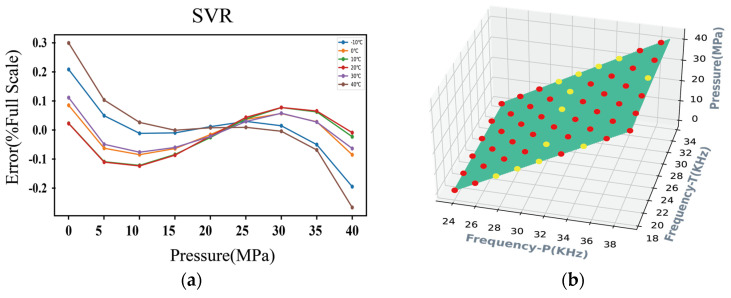

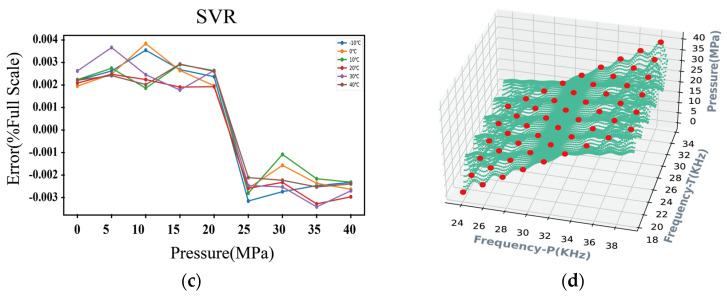
Performance of SVR for varying values of parameter γ. (**a**,**b**) illustrate the predictive accuracy and generalization ability for γ= 1 × 10^−4^, respectively, and those for γ=10 are illustrated in (**c**,**d**), respectively. Calibration data is indicated by the yellow dots, and the green areas depict the three-dimensional graphs of the regression function. The support vectors of the SVR model are represented by the red dots.

**Figure 16:**
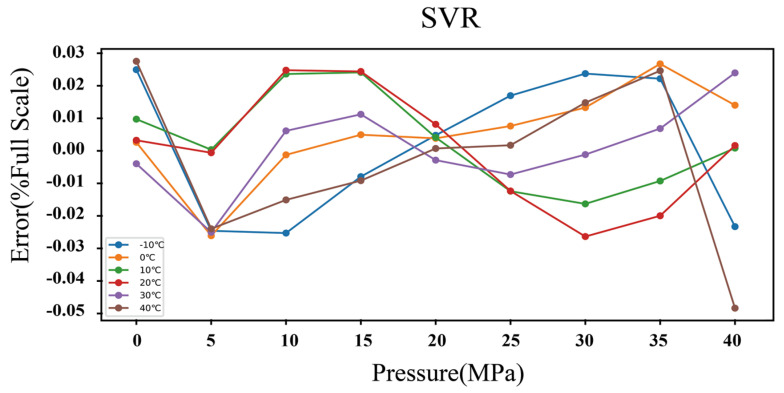
Accuracy of the resonant pressure sensor based on the SVR temperature compensation model.

**Figure 17:**
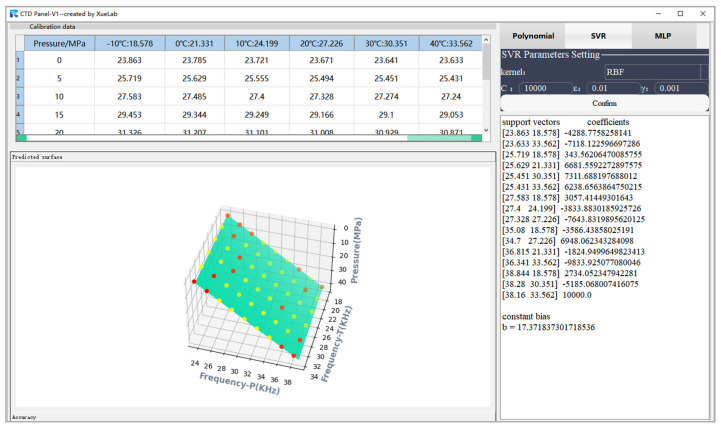
Interface diagram of the CTD Panel tool for building a temperature compensation model of SVR for the sensor. Calibration data is indicated by the yellow dots, and the green areas depict the three-dimensional graphs of the regression function. The support vectors of the SVR model are represented by the red dots.

The scenarios corresponding to γ= 1 × 10^−4^ and 10 are shown, respectively, in Figure 15, which illustrates the influence of parameter γ on the construction of the SVR model. In scenario 1, depicted in the top part of the graph, the derived model with γ = 1 × 10^−4^ guarantees a good generalization performance at the expense of predictive accuracy, which is equal to ±0.3% FS. In scenario 2, depicted in the bottom part of the graph, the SVR model with γ= 10 may be overfitting despite the high accuracy of ±0.004%FS obtained on the training examples. After experimenting with various manual hyperparameter settings, the optimal performance is achieved with the following values: C = 1 × 10^4^, ε = 0.01, and γ = 1 × 10^−3^. Figure 16 shows the validation accuracy of the model, where an accuracy of ±0.05%FS can be observed. The location of the optimal hyperplane in the data space is determined by the 16 data points selected as support vectors, as shown in Figure 17, which means that the solution vector is sparse. Finally, the nonlinear regression model can be defined according to the following expression:

(24) $$f^{*}\left(x\right)=\sum_{i=1}^{16}{\alpha_{i}}^{\left(*\right)}e^{-\gamma \left[{\left(x_{i}^{\left(1\right)}-x^{\left(1\right)}\right)}^{2}+{\left(x_{i}^{\left(2\right)}-x^{\left(2\right)}\right)}^{2}\right]}+b$$

### 3.5. Comparison of Generalization for the Three Predominant Regression Methods

The generalization performance of a learning method is evaluated by its prediction capacity on a set of unseen samples drawn from a distribution identical to that of the training set. With $f^{*}$, i.e., MPR, SVR, and MLP, being an alternative estimator of the response y given the regressor x, the estimation error between the output of the learning model $f^{*}$ and the ground-truth y averaged over the data distribution and datasets can be used to assess the model’s performance. The error is calculated according to (25) as follows:

(25) $$E_{g}=\frac{1}{N}\sum_{n=1}^{N}{\left(f^{*}\left(x_{n}\right)-y_{n}\right)}^{2}$$

**Figure 18:**
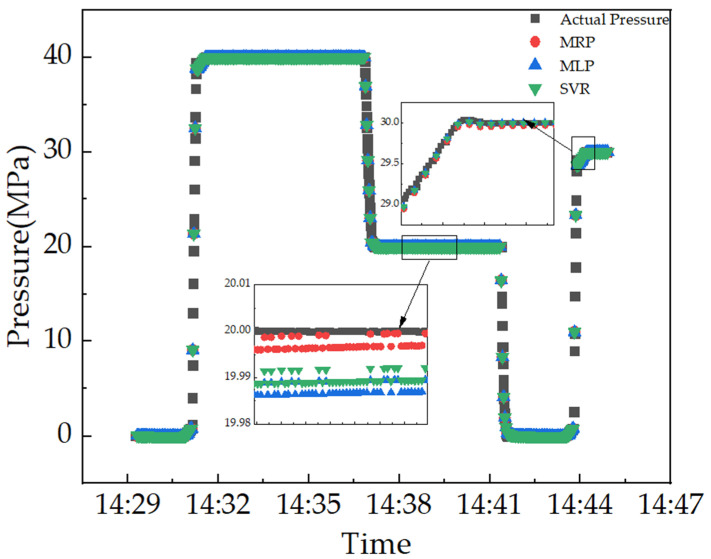
A comparison of three different learning methods for predicting pressure.

In the dynamic test experiments conducted in this study, as shown in Figure 18, the real pressure information is obtained from a standard pressure measurement instrument for a relatively short time interval, whereas the frequency measurement is performed every two seconds by the data acquisition device. There is no way to precisely calculate the estimation error because the actual pressure corresponding to each observation of the sensor’s frequency output is unknown. Nevertheless, a direct analysis of the graph demonstrates that the generalization capacity of MPR in a dynamic test is relatively better, which is sufficient for meeting the specified goals of the application of interest in the real world.

## 4. Discussion

The MRP method provides an optimal estimator in the minimum Euclidean distance sense by solving the LS problem. This numerical analysis algorithm achieves a superb accuracy of 0.008% FS for the fabricated pressure sensor, which is equivalent to a maximum absolute error of 0.0032 MPa. It is impressive compared to the preliminary measurement error of 1.8 MPa validated in Section 3.1. The performances of these three estimators are summarized in Table 1:

The MLP method for temperature compensation is indeed suboptimal, with a relatively low accuracy of 0.3% FS because the ANN’s architecture is configured empirically and there are limited training examples. However, its important virtue is the broadened application of discovering the most intricate relationship and performing highly complex tasks based on the intuitive parallel distributed network. Furthermore, the finite scalar multiplication arithmetic renders it a highly useful computational asset for the pressure calibration process.

Support vector regression, the representative of statistical learning, has a sparse solution space and is insensitive to anomalous observations. In this paper, it exhibits effective performance with a high accuracy of 0.05% FS. However, its computational complexity is exponential vis-a-vis the kernel operation, which renders it inappropriate for real-time problems involving large datasets. 

## 5. Conclusions

In this paper, AT-cut quartz was used as the core of the resonant sensor for high-range pressure sensing. Despite its intrinsic high precision, its nonlinear characteristic associated with the varying temperature limited its applicability in many potential applications. In order to eliminate the erroneous effect of temperature on sensor response, three predominant learning paradigms for temperature compensation in resonant pressure sensors were described. These benchmark algorithms for temperature compensation have been determined through the analysis of experimental data within the temperature range of −10–+40 °C and pressure range of 0–40 MPa.

The prediction accuracy of the MPR model relies heavily on its order as it dictates the dimensions of the projected polynomial feature space. We optimized the best order for the design matrix to be 5. The residual error between −10 °C and 40 °C was 0.008% FS. In the MLP model, to achieve a compromise between global optimization and model complexity, we incorporated two hidden layers in the networks. Accordingly, five and two neurons were allocated to the first and second hidden layers, respectively. In order to combat the issue of vanishing gradient, the nonlinear activation function chosen for hidden layers is ReLU. Upon the culmination of 2000 training epochs, the model proves its ability to enhance the sensor’s accuracy to 0.3% FS. In the SVR model, the RBF kernel was enriched with the inclusion of the receptive filed terminology, and, by following this guiding principle, we have ultimately ascertained the most ideal values for parameters C, ε, and γ to be 1 × 10^4^, 0.01, and 1 × 10^−3^, respectively. With the adoption of this novel approach, the sensor’s accuracy has been improved to an impressive level, achieving a deviation of 0.15% in the full-scale range.

With respect to computational complexity and predictive accuracy, we asserted that the multivariate polynomial regression (MPR) method provided the practical basis for the optimal design of nonlinear systems for temperature compensation. With its high accuracy of 0.008% FS and remarkable generalization performance, the MPR proved to be the ideal choice for our task.

## Figures and Tables

**Table 1:** An overview of the performance of MPR, MLP, and SVR for temperature compensation in a quartz resonant pressure sensor.

Method	Maximum Absolute Error (MPa)	The Residual Error at all Temperatures of Full Scale	Rank of Generalization Ability
MPR	0.0032	0.008% FS	High
MLP	0.12	0.3% FS	Low
SVR	0.02	0.05% FS	Middle

## Data Availability

Data are contained within the article.
